# Opioid administration guided by Surgical Pleth Index in patients with a combination of general and regional anaesthesia during trauma and orthopaedic surgery: a double-blind, randomised controlled trial

**DOI:** 10.1007/s10877-025-01363-2

**Published:** 2025-10-03

**Authors:** Kim C. Koschmieder, Hans O. Pinnschmidt, Lea-Sophie Borst, Gillis Greiwe, Elena Kainz, Marlene Fischer, Rainer Nitzschke

**Affiliations:** 1 Department of Anesthesiology, Center of Anesthesiology and Intensive Care Medicine, University Medical Center Hamburg-Eppendorf, Hamburg, Germany; 2 Institute of Medical Biometry and Epidemiology, University Medical Center Hamburg-Eppendorf, Hamburg, Germany

**Keywords:** Regional anaesthesia, Opioid analgesics, Nociception, Depth of anaesthesia, Monitoring - intra-operative, Pain measurement.

## Abstract

**Purpose:**

This randomised controlled trial investigated the effect of Surgical Pleth Index (SPI) guided sufentanil administration on intraoperative sufentanil consumption compared to routine care in patients with a combination of general anaesthesia and regional anaesthesia having trauma and orthopaedic surgery.

**Methods:**

Eighty patients with a combination of general anaesthesia and regional anaesthesia undergoing trauma or orthopaedic surgery were randomised into two groups to receive either sufentanil guided by SPI monitoring or by routine care (Control). The primary endpoint was intraoperative sufentanil consumption. Secondary endpoints were postoperative pain level, opioid consumption, incidence of nausea, duration of time in the post-anaesthesia care unit (PACU) and quality of recovery.

**Results:**

The median intraoperative sufentanil administration adjusted to bodyweight and surgery duration did not differ between the groups: SPI guided group 2.29 (interquartile range, IQR 0.29 to 6.91), Control 1.65 (IQR 0.83 to 2.63) µg·kg^−1^·min^−1^*1000 (*P* = 0.906). The relative risk for receiving intraoperative sufentanil was RR 0.909 (95% CI 0.723 to  1.143, *P* = 0.414). Median morphine equivalents administered in the 24 h after discharge from the PACU were 3.8 (IQR 0.0 to 22.5) in the SPI guided group and 19.1 (IQR 3.8 to 30.0) mg (*P* = 0.021) in the control group without adjustment for multiple testing. Other secondary endpoints showed no differences.

**Conclusion:**

SPI guided sufentanil administration did not reduce intraoperative sufentanil consumption compared to routine care in patients having trauma and orthopaedic surgery with a combination of general anaesthesia and regional anaesthesia.

**Trial registration:**

Clinicaltrials.gov identifier NCT06040307 (registered September 8, 2023).

## Introduction

The goal of the administration of opioids during general anaesthesia is anti-nociception, that facilitates reflex control of the sympathetic nervous system, immobility of the surgical field without defensive movements, absence of pain and a fast postoperative recovery [1, 2]. Yet, opioids have severe side effects and may cause arterial hypotension, bradycardia, prolonged postoperative recovery, delirium, somnolence, nausea and vomiting, urinary retention, pruritus, reduced gastrointestinal motility and respiratory depression [1, 2]. Not least, anaesthesiologists must bear in mind that unreflected opioid prescription led to the opioid epidemic in Anglo-American countries [3]. For many patients who later develop an opioid use disorder, the initial opioid exposure took place in the perioperative period [3]. Thus, modern general anaesthesia aims to avoid an overdose of opioid analgesics and reduce opioid administration with different techniques [3–5].

To achieve intraoperative anti-nociception and postoperative analgesia, general anaesthesia is often combined with peripheral regional anaesthesia [6]. However, the effectiveness of regional anaesthesia conducted during general anaesthesia is difficult to evaluate. A peripheral anaesthesia of the lower extremities may, at times, provide incomplete coverage of the surgical field, necessitating supplemental intraoperative opioid administration. Beyond the inherent anatomical limitations of lower extremity nerve blocks, the success of regional anaesthesia depends heavily on provider experience and may occasionally fail completely. When regional anaesthesia is combined with general anaesthesia, intraoperative assessment of block effectiveness remains difficult, making it challenging to tailor opioid analgesia. Consequently, prophylactic or empiric opioid administration remains common practice during combined general and regional anaesthesia to mitigate inadequate nociception control.

During general anaesthesia, anaesthesiologists traditionally choose the intraoperative dose of opioids according to changes of heart rate, blood pressure, patient’s movements, sweating and tears [7]. In recent years, different monitoring devices estimating the effect of nociception during general anaesthesia have become commercially available. These monitoring devices index the nociception/analgesia balance and should assist anaesthesiologists choosing the adequate dose of opioid analgesics during general anaesthesia [8, 9]. One of the first nociception indices was the Surgical Pleth Index (SPI) derived by the CARESCAPE™B650 patient monitor (GE Healthcare, Helsinki, Finland) [10–13]. An index value is defined from normalised heart rate and pulse wave amplitude derived by photoplethysmography [8].

The study tested the H1 hypothesis, that SPI guided sufentanil administration reduces intraoperative sufentanil consumption in total and adjusted per kg bodyweight and duration of surgery, compared with routine care in patients with a combination of general anaesthesia and regional anaesthesia. Secondary aims were to investigate whether SPI guided sufentanil administration reduces postoperative pain level and morphine consumption or improves postoperative recovery.

## Methods

This study was a prospective interventional clinical trial that randomised patients to an intervention or a control group during general anaesthesia. The study was approved by the regional ethics review board (Medical Council of Hamburg, Germany, reference number 2023–101127-BO-ff, chairperson Torsten Christ) on August 18, 2023 and registered with clinicaltrials.gov (NCT06040307, date of registration: September 8, 2023) before patient recruitment. Participating patients gave written informed consent at least one day before surgery. The manuscript adheres to the applicable CONsolidated Standards Of Reporting Trials (CONSORT) guidelines.

### Inclusion and exclusion criteria

The study team screened all patients scheduled for trauma or orthopaedic surgery with a combination of general and regional anaesthesia for eligibility. All adult patients (> 18 year) undergoing elective trauma or orthopaedic surgery with a general anaesthesia in combination with a regional anaesthesia were eligible for inclusion. Exclusion criteria were preoperative treatment with beta-receptor blocking agents or digitalis, cardiac pacemaker, high degree cardiac arrhythmias (including atrial fibrillation), severe peripheral or cardiac neuropathy, intraoperative treatment with ketamine, beta-receptor agonists or clonidine, preoperative chronic opioid abuse, the patient’s inability to specify postoperative pain level, and a postoperative intensive care unit stay.

### Randomisation

The investigators randomly assigned the study patients into a SPI guided group or a control group by a sealed envelope system. The randomisation sequence was generated by a physician not involved in the study before patient recruitment via a computer-generated list using the RANDBETWEEN function in Microsoft Excel (Microsoft Corporation, Redmond, WA). Allocation was concealed with sequentially numbered sealed opaque envelopes. The principal investigator evaluated eligibility, a physician of the study staff obtained informed consent, and the participants were enrolled by opening the respective concealed envelope containing the patient allocation immediately before anaesthesia induction.

### Anaesthesia

All patients received preoperative care following institutional standards in all groups. Prior to anaesthesia induction, convective air warming and routine monitoring including 5-lead electrocardiography, non-invasive blood pressure monitoring and pulse oximetry (IntelliVue MX500, Philips Healthcare, Amsterdam, Netherlands) were installed. All patients received a general anaesthesia either as a balanced anaesthesia with sevoflurane and sufentanil or as a total intravenous anaesthesia with continuous propofol infusion and sufentanil. For the induction of general anaesthesia, the patients received a standardised sufentanil bolus of 0.3 µg·kg^−1^ bodyweight (BW) followed by a propofol bolus of 2 mg·kg^−1^ BW. The airway was secured with either a laryngeal mask or a tracheal tube after rocuronium bromide 0.5 mg·kg^−1^ was administered for tracheal intubation. After induction of general anaesthesia capnography, bispectral index (BIS) monitoring (BIS OEM-Modul, Medtronic, Dublin, Ireland) (with a target range of BIS values between 40 and 60), acceleromyography for train-of-four (TOF) counts (Philips Intellivue NMT module, Philips Healthcare, Amsterdam, Netherlands) and body temperature measurement were implemented.

Dexamethasone 4 mg and ondansetron 4 mg were administered to all patients for prophylaxis of postoperative nausea and vomiting (PONV) as per institutional routine. Patients received a peripheral regional anaesthesia after the induction of general anaesthesia with ultrasound-guided (TE7, mindray, Shenzhen, China) injection of ropivacaine. For surgery on the lower limb inguinal femoral block, adductor canal block, or sciatic nerve block was performed with 0.75% ropivacaine. For surgery on the upper limb, regional anaesthesia consisted of a brachial plexus block from the interscalene, supraclavicular or axillary approach with 0.1% or 0.2% ropivacaine, according to institutional standards. The decision to use 0.1% or 0.2% ropivacaine (20 to 30 ml) for a brachial plexus block was a pre-operative case-to-case team decision between anaesthesiologists and surgeons.

Patients received continuous norepinephrine administration if necessary to maintain the mean arterial pressure above 65 mm Hg. Intraoperative sufentanil was administered as described in the study protocol. Metamizole 1000 mg was administered for pre-emptive analgesia during general anaesthesia before the end of surgery.

### Study protocol

In the SPI guided group, intraoperative administration of sufentanil was guided by SPI monitoring. The monitoring device normalises the photoplethysmographic amplitude (PPGA) and the heartbeat interval (HBI) derived from finger photoplethysmography and calculates the SPI using the following equation: SPI = 100 − (0.7 × PPGAnorm + 0.3 × HBInorm). The SPI is represented on a scale from 0 (low sympathetic tone) to 100 (high sympathetic tone) and the proposed target of SPI values during surgery is between 20 and 50 [8]. The monitoring device does not require any disposable equipment. In the present study, 5 µg sufentanil were administered if SPI value was above 50 for more than 30 s. Administration of 5 µg sufentanil was repeated every 5 min without a predefined maximum dose until SPI value remained under 50 again.

In the control group, intraoperative sufentanil administration was left at the discretion of the attending anaesthesiologist using clinical signs based upon changes of heart rate, blood pressure, lacrimation, sweating and spontaneous movements of the patient. As orientation for sufentanil administration, anaesthesiologists were recommended to administer 5 µg sufentanil if systolic blood pressure was > 140 mmHg or mean blood pressure was > 100 mmHg or heart rate was > 90/min. If deemed necessary, the attending anaesthesiologists were allowed to administer higher or lower sufentanil doses at their discretion.

All patients were transferred to the postanaesthesia care unit (PACU) for standard institutional postoperative care. In the PACU, the patient’s pain level was assessed on a numerical pain rating scale (NRS) from 0 to 10 by a member of the study team every 15 min once the patients were able to effectively communicate. Patients received 3.75 mg piritramide (corresponding to 2.5 mg morphine equivalents) if NRS score was > 3/10. The fit-for-discharge criteria from the PACU were fulfilled when the modified Aldrete Score reached 8.

After discharge from the PACU, all patients received a standardized stepwise analgesic ladder for pain management. If rated necessary, first-line analgesia consisted of oral metamizole 1000 mg on a fixed schedule every 6 h and additional oral ibuprofen 400 mg on-demand. Second step analgesia consisted of oral oxycodone 5 mg with retarded release (corresponds to 7.5 mg morphine) and oral non-retarded oxycodone 5 mg on demand. For further demand of pain therapy IV piritramide 7.5 mg was used.

### Measurements and data handling

The intraoperative data were documented by a study assistant, who was not involved in the patient’s treatment. During surgery, in the SPI guided group the study setting precluded blinding of the attending anaesthesiologist. Although the anaesthesiologists in the operation theatre were not part of the study team, they were instructed to strictly follow the study protocol whenever the patients had been randomised to the SPI guided group. In the control group, the anaesthesiologists in the operation theatre were blinded to the SPI monitor screen and, if possible, blinded to the purpose of the study. In this study, the patients, the study team in the PACU, physicians and nurses in the PACU were blinded to group assignment. During the PACU stay, a blinded member of the study team assessed the data on postoperative pain and consumption of analgesics, nausea and vomiting, shivering, or other anaesthesia-related events. Twenty-four hours after discharge from the PACU, patients were asked to rate their pain level at rest by a member of the study team who was blinded to the group assignment. At the same time, patients were also interviewed for their postoperative quality of recovery using the Quality-of-Recovery (QoR-15) Score with a minimum of 0 and a maximum of 150 values [14, 15].

### Primary and secondary endpoints

The two co-primary endpoints were the intraoperative total amount of sufentanil as well as the intraoperative sufentanil consumption adjusted to kg body weight and surgery duration. The secondary endpoints were cumulative intraoperative norepinephrine consumption, duration between end of surgery until extubation or removal of laryngeal mask, immediate postoperative pain, highest postoperative pain level and mean postoperative pain level at the PACU, morphine equivalents administered in the PACU, incidence of postoperative nausea and vomiting, duration of time in the PACU until fit for discharge, mean pain level and morphine equivalents administered 24 h after discharge from the PACU and postoperative QoR-15 Score. The study investigated associations between the total amount of sufentanil, the total amount of ropivacaine and the SPI value before skin incision and postoperative pain and opioid consumption.

### Sample size calculation

Pre-test data in a consecutive sample of routine patients with a combination of general and regional anaesthesia during trauma and orthopaedic surgery in the authors institution showed a mean ± SD intraoperative sufentanil dose of 25.5 ± 10.7 µg. The study was designed to detect a reduction of the intraoperative sufentanil consumption by 30% (8 µg). Based on these data, sample size calculations were performed using PASS 2008 (version 08.0.6, NCSS LLC, Kaysville, UT, USA). To achieve 95% power in detecting a significant reduction in sufentanil consumption in the SPI-guided group — assuming a mean of 17.5 µg versus 25.5 µg in the control group, with a shared standard deviation of 10.7 µg — a sample size of 40 patients per group was required. Calculations for the two co-primary endpoints were performed using a one-sided two-sample t-test with a significance level of 0.05.

### Statistical analyses

Patient, surgery and anaesthesia characteristics are presented as mean (standard deviation) and absolute numbers (%). The progression of SPI values over time in the two study groups were compared using a longitudinal linear mixed model with random intercepts for patients, accounting for repeated measures within patients and assuming the study group allocation and time as fixed effects. Data on intraoperative sufentanil consumption were right-skewed as assessed via histograms. Data of primary and secondary endpoints are presented with median and interquartile range or frequency with percentage. Group differences were evaluated using the Mann-Whitney-U test or Fisher’s exact test, as appropriate. The authors calculated the relative risks for receiving intraoperative sufentanil as well as opioids in the 24 h after discharge from the PACU using the SPSS routine GENLIN, assuming a binomial data distribution and applying a log link function. Spearman’s rank correlation coefficients were calculated for correlations between the total amount of sufentanil and the total amount of ropivacaine used for regional anaesthesia on the one hand and postoperative pain and opioid consumption on the other hand. *P* < 0.05 in a one-sided test was considered significant for the co-primary endpoints. Secondary objectives were considered purely exploratory, without adjusting the significance level for multiple testing. Statistical analyses were performed using SPSS 29.0.1.0 (IBM SPSS Statistics Inc., Armonk, NY, USA).

## Results

**Fig. 1:**
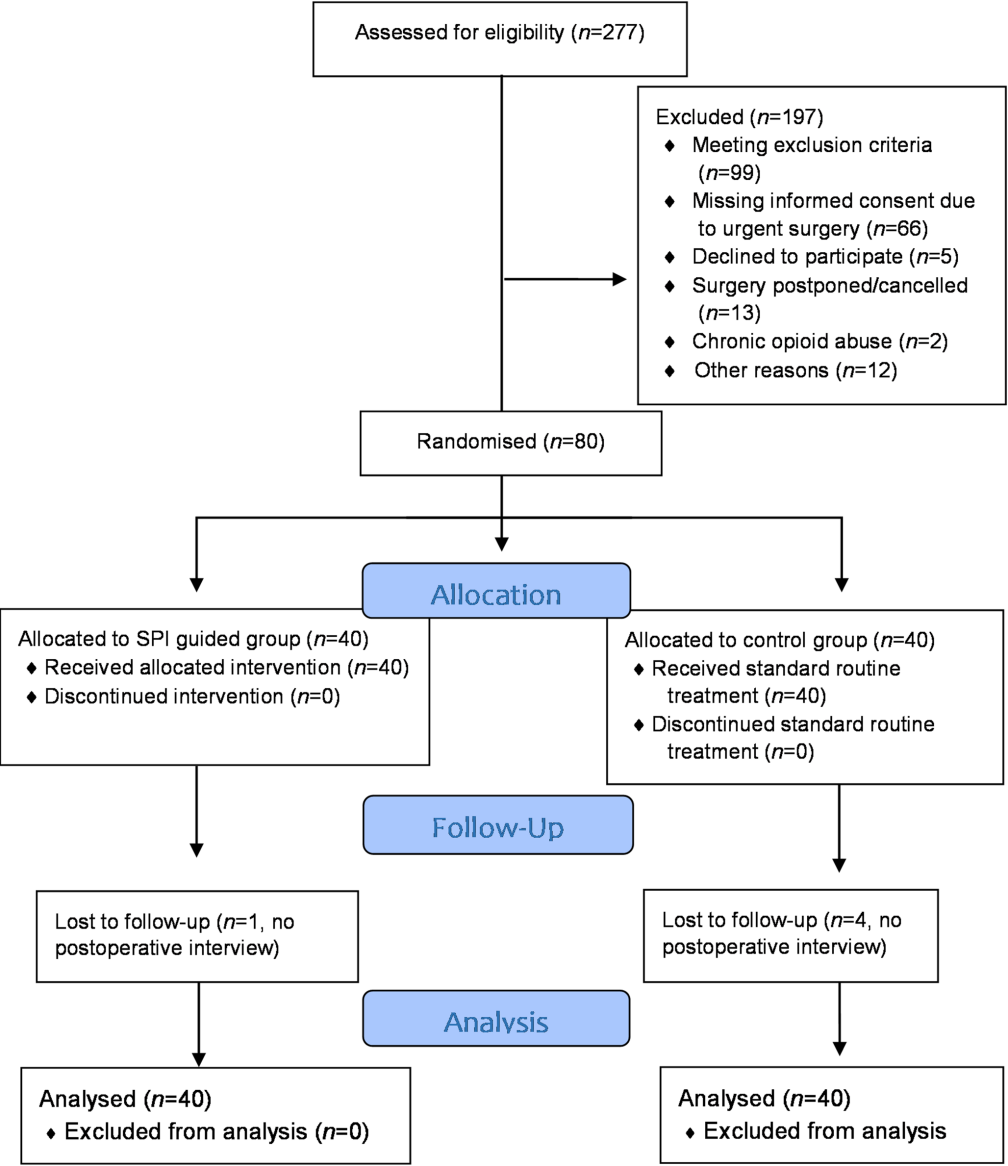
CONSORT (CONsolidated Standards Of Reporting Trials) diagram, Study flow chart

The study staff screened 277 patients scheduled for trauma or orthopaedic surgery with a general anaesthesia that was combined with a regional anaesthesia in the operation theatre where the study was conducted between December 18th 2023 and April 9th 2024. Figure 1 displays the CONSORT flow diagram, including the details of assessment and exclusion. After obtaining written informed consent, 80 patients were randomised to either the SPI guided group or the control group (40 each). Biometric data, surgery and anaesthesia characteristics are displayed in Table 1.

**Fig. 2:**
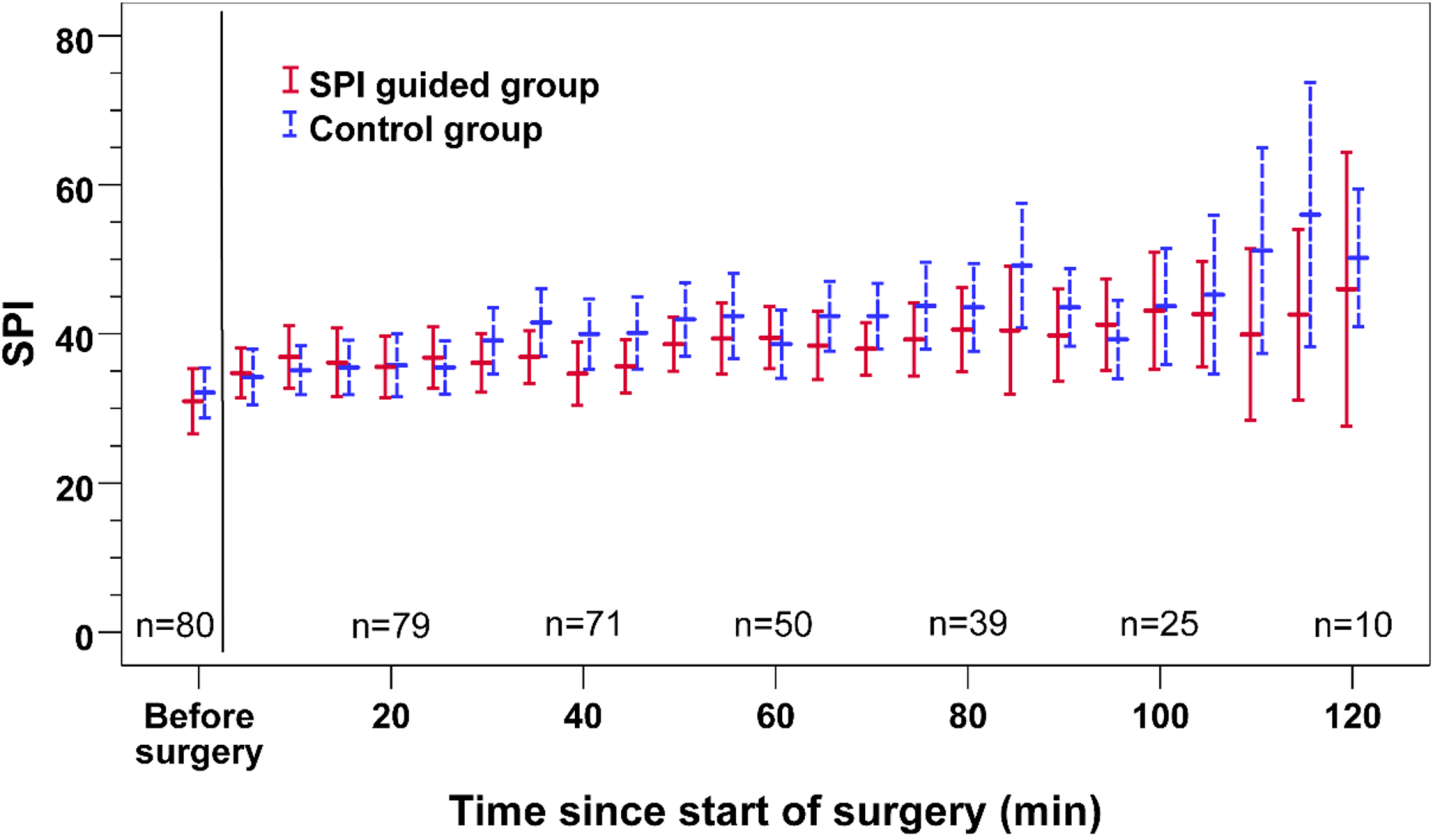
The course of intraoperative SPI values during the first 120 Minutes of the operation in the study and control group

Figure 2 shows the mean SPI values and 95% CI over time during the first 2 h of surgery in the SPI guided and the control group. Towards the end of observation, confidence intervals increase due to declining patient numbers caused by differences in the length of surgery. Mixed model analysis revealed that SPI values significantly increased over the course of the operation at a rate of 0.081 · min^−1^ (95% CI 0.056 to 0.105; *P* < 0.001), but there was no significant difference in SPI values between groups.

**Fig. 3:**
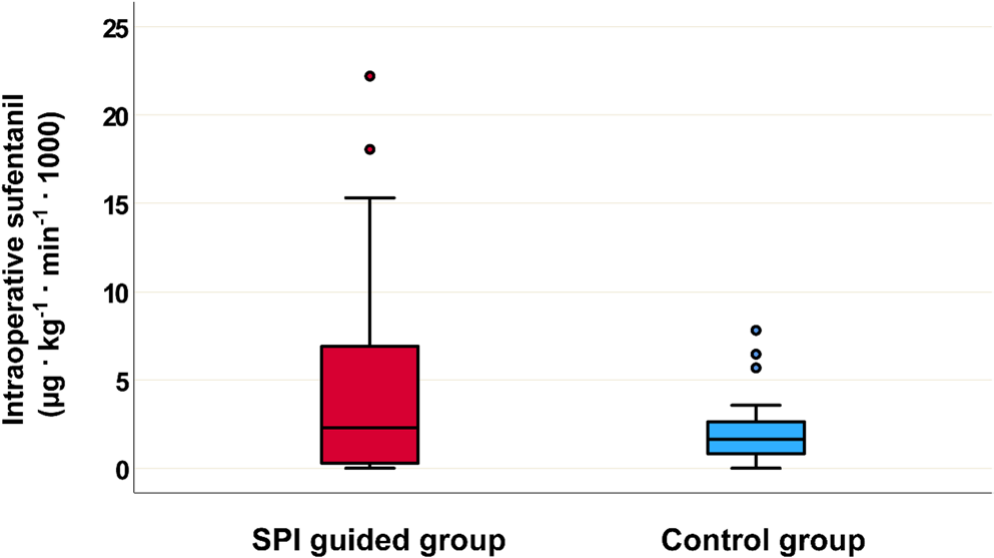
Distribution of intraoperative sufentanil dose adjusted for the patients’ body weight and duration of anaesthesia in the SPI guided group and in the control group as the primary endpoint. The data are presented as medians, ranges, and interquartile ranges (25th–75th percentile)

The co-primary endpoints investigated intraoperative total sufentanil consumption and intraoperative sufentanil consumption adjusted for body weight and duration of surgery. Table 2 shows that neither intraoperative total sufentanil consumption nor intraoperative adjusted sufentanil consumption were significantly lower in the SPI guided group, as compared with the control group. After anaesthesia induction, patients received a median sufentanil dose of 2.29 (IQR 0.29 to 6.91) µg · kg^−1^ · min^−1^ *1000 in the SPI guided group and 1.65 (IQR 0.83 to 2.63) µg · kg^−1^ · min^−1^ *1000 in the control group per kg body weight and surgery duration (*P* = 0.906) (Fig. 3). In the SPI guided group, 10 (25%) patients did not receive any sufentanil during surgery, while in the control group 7 (17.5%) patients did not receive intraoperative sufentanil. The relative risk for receiving intraoperative sufentanil was RR 0.909 (95% CI 0.723 to  1.143, *P* = 0.414).

Within the secondary endpoints there was a trend towards less postoperative morphine equivalents administered after discharge from the PACU in the SPI guided group with a median dose of 3.8 (IQR 0.0 to 22.5) µg in the SPI guided group compared with 19.1 (IQR 3.8 to 30.0) µg in the control group (*P* < 0.021, Table 2). The relative risk to receive an opioid in the 24 h after discharge from the PACU was RR = 0.667 (95% CI 0.466 to 0.953, *P* = 0.026).

Data analysis did not reveal significant correlations between the total amount of intraoperative sufentanil and the total amount of ropivacaine used for regional anaesthesia on the one hand and postoperative pain and opioid consumption in the PACU and in the first 24 h after discharge from the PACU on the other hand (Table 3).

**Table 1:** Patient, surgery and anaesthesia characteristics of the study groups

	SPI guided group (*n* = 40)	Control group (*n* = 40)
**Biometric data** Age [years]	49 ± 17	48 ± 17
Female [*n*]	17 (42,5%)	20 (50%)
Height [cm]	175 ± 10	175 ± 9
Weight [kg]	85 ± 21	77 ± 15
BMI [kg·cm^−2^]	28 ± 6	25 ± 5
ASA class		
- I	16 (40%)	17 (42.5%)
- II	17 (42.5%)	17 (42.5%)
- III	7 (17.5%)	6 (15%)
Preoperative Medication		
Metabolic	5 (13%)	3 (8%)
Anticoagulants	3 (7.5%)	8 (20%)
Antihypertensive	9 (23%)	5 (13%)
Non-opioid analgesics	14 (35%)	16 (40%)
Opioids	2 (5%)	6 (15%)
No medication	14 (35%)	12 (30%)
NRS before surgery	1.3 ± 1.7	1.2 ± 1.7
**Type of surgery**		
Ankle joint osteosynthesis	5 (12.5%)	6 (15%)
Arthroscopic knee surgery	8 (20%)	6 (15%)
Humerus osteosynthesis	0 (0%)	2 (5%)
Radius osteosynthesis	7 (17%)	8 (20%)
Arthroscopic shoulder surgery	2 (5%)	4 (10%)
Elbow osteosynthesis	8 (20%)	3 (8%)
Lower extremity corrective osteotomy	1 (3%)	4 (10%)
Total shoulder arthroplasty	1 (3%)	3 (8%)
Others	8 (20%)	4 (10%)
Surgery duration [min]	83 ± 37	83 ± 45
Intraoperative use of a tourniquet	26 (65%)	23 (56%)
**Type of anaesthesia**		
TIVA	25 (62.5%)	29 (72.5%)
Balanced anaesthesia	15 (37.5%)	11 (27.5%)
Dose of ropivacaine [mg]	124 ± 84	115 ± 73
Mean BIS value	41 ± 6	42 ± 6
Body temperature at the end of surgery [°C]	36.3 ± 0.52	36.4 ± 0.49
Infusion quantity [ml]	1014 ± 711	880 ± 266
Anaesthesia duration [min]	146 ± 41	149 ± 51

Continuous data are presented as mean ± SD and categorical data are presented as absolute numbers (%). BMI, body mass index; ASA, American Society of Anesthesiologists physical status; NRS, numeric pain rating scale; TIVA, total intravenous anaesthesia.

Linear mixed model analysis revealed a significant effect of time on SPI values with a mean increase in SPI values of 0.81 (95% CI 0.56 to 1.05; *P* < 0.001) every 10 min, while there was no evidence found for a significant effect of group allocation (*P* = 0.463). Error bars show estimated marginal means with 95% CI. Red solid bars = SPI guided group, blue dashed bars = Control group.

**Table 2:** Primary and secondary endpoints

	SPI guided group (*n* = 40)	Control group (*n* = 40)	*P*-Value	
**Primary endpoint**				
Intraoperative sufentanil consumption [µg]	15.0 (IQR 2.5 to 40.0)	10.0 (IQR 5.0 to 18.75)	0.834	
Intraoperative sufentanil consumption per kg body weight and surgery duration [µg · kg^−1^ · min^−1^ *1000]	2.29 (IQR 0.29 to 6.91)	1.65 (IQR 0.83 to 2.63)	0.906	
**Secondary endpoints**				
Cumulative dose of norepinephrine consumption [µg]	390.5 (IQR 225.5 to 908.5)	321.5 (IQR 184 to 723)	0.707	
Time from end of narcotics to extubation or LMA removal [min]	9.5 (IQR 7 to 15)	9 (IQR 5 to 12.5)	0.245	
Immediate postoperative pain level at arrival in the PACU [NRS]	0.0 (IQR 0 to 0)	0.0 (IQR 0 to 3)	0.158	
Highest postoperative pain level at the PACU [NRS]	0.0 (IQR 0.0 to 4.5)	1.0 (IQR 0.0 to 5.])	0.538	
Mean postoperative pain level at the PACU [NRS]	0.0 (IQR 0.0 to 1.8)	0.7 (IQR 0.0 to 3.4)	0.339	
Incidence of moderate to severe postoperative pain (NRS 4–10) at the PACU	14 (35%)	13 (33%)	1.000	
Incidence of PONV at the PACU	4 (10%)	2 (5%)	0.675	
Duration of time in PACU until fit-for-discharge status [min]	40.0 (IQR 29.5 to 47.5)	30.5 (IQR 26.0 to 43.0)		0.067
Morphine equivalents administered in the PACU [mg]	0.0 (IQR 0.0 to 2.6)	0.0 (IQR 0.0 to 3.0)		0.545
Postoperative pain level at rest 24 h after the operation [NRS]	3 (IQR 2 to 6)	4 (IQR 2 to 7)		0.492
Morphine equivalents administered in the 24 h after discharge from the PACU [mg]	3.8 (IQR 0.0 to 22.5)	19.1 (IQR 3.8 to 30.0)		0.021
Quality of recovery 24 h after the operation [QoR-15-Score]	113 (IQR 96 to 120)	107 (IQR 90.5 to 121.5)		0.500

Data are presented as median (IQR) or absolute number (percentage). P-values of the co-primary endpoints were based on a one-sided MWU test and P -values of the secondary endpoints were based on a two-sided MWU test. SPI, Surgical Pleth Index; IQR, interquartile range; NRS, Numeric rating scale (from 0= ‘no pain’ to 10 = ‘worst pain’); PACU, postanaesthetic care unit; PONV,postoperative nausea and vomiting; QoR-15, 15-item Quality-of-Recovery Score (from0 = ’poor outcome’ to 150 = ’excellent outcome’).

**Table 3:** Correlations between the total amount of sufentanil and the total amount of ropivacaine and postoperative pain and opioid consumption

Independent Variable	Correlation with the mean postoperative NRS in the PACU	Correlation with the highest postoperative NRS in the PACU	Correlation with the dose of morphine equivalents in the PACU	Correlation with the NRS at rest 24 h after discharge from the PACU	Correlation with the dose of morphine equivalents in the first 24 h after discharge from the PACU
Total amount of sufentanil [µg]	ρ= − 0.005 (*P* = 0.964)	ρ= − 0.019 (*P* = 0.866)	ρ= − 0.001 (*P* = 0.996)	ρ= − 0.027 (*P* = 0.819)	ρ = 0.140 (*P* = 0.217)
Total amount of ropivacaine [mg]	ρ= − 0.123 (*P* = 0.279)	ρ= − 0.075 (*P* = 0.509)	ρ= − 0.147 (*P* = 0.192)	ρ= − 0.044 (*P* = 0.706)	ρ = 0.069 (*P* = 0.543)

NRS, Numeric rating scale; PACU, postanaesthetic care unit; ρ, Spearman rank correlation coefficient

## Discussion

The present study did not confirm the hypothesis that SPI guided sufentanil administration reduces intraoperative sufentanil consumption compared with routine care in patients with a combination of general anaesthesia and regional anaesthesia undergoing trauma or orthopaedic surgery and the null hypothesis could not be rejected. During the follow-up period, no difference was observed in recovery times (time to extubation and time in the PACU until fit for discharge), postoperative pain level and morphine consumption in the PACU and 24 h after discharge from the PACU, the incidence of PONV, and postoperative quality of recovery. The risk to receive any opioid in the 24 h after discharge from the PACU was lower in the SPI guided group than in the control group. Yet, this trend towards less postoperative morphine equivalents administered after discharge from the PACU in the SPI guided group was not considered statistically significant taking into account the number of multiple secondary endpoints tested in an explorative manner without adjustment for multiple testing. Besides, our results demonstrate that neither the dose of intraoperative sufentanil nor the dose of ropivacaine used for regional anaesthesia showed an association with the level of postoperative pain and postoperative opioid consumption.

To account for the many adverse effects of opioids, various opioid-sparing strategies have been proposed [4, 16]. Combining general anaesthesia with surgical site-specific regional anaesthesia is one of the strategies that effectively reduce perioperative pain and decrease the administration of opioids [4, 6, 17, 18]. SPI monitoring has been validated in assessing the effectiveness of regional anaesthesia in the unconscious patient and SPI values were lower in patients with interscalene plexus block and abdominal wall blocks compared to patients with general anaesthesia only (18, 19). But while regional anaesthesia as a non-opioid perioperative analgesic strategy reduces opioid consumption during surgery, our results show that opioid titration guided by SPI monitoring with a target range between 20 and 50 did not reduce opioid consumption in patients with a combination of general anaesthesia and regional anaesthesia compared with routine care. In this regard, it is not clear if SPI monitoring accurately reflects nociception in patients with a combination of general anaesthesia and regional anaesthesia.

The fact that there was no difference in SPI values and in opioid consumption between the attending anaesthesiologists in the control group on the one hand and the SPI monitoring in the SPI guided group on the other hand suggests that in both study groups the analgesia-nociception balance was assessed similarly (Fig. 2; Table 2). As the SPI is calculated from normalised heart rate and pulse wave amplitude it is derived solely from haemodynamic parameters under the influence of sympathetic tone. In the same way, in the traditional way of choosing the necessary intraoperative opioid dose anaesthesiologists mainly rely on the interpretation of changes in heart rate and blood pressure. The present results suggest that guidance of opioids by nociception monitoring based solely on haemodynamic parameters is no better than opioid administration guided by the discretion of the attending anaesthesiologist. In either way, haemodynamic changes due to the sympathetic tone lead to the dose of opioids administered. In the setting of this study, opioid guidance by SPI monitoring did not result in a clinical benefit. SPI monitoring might not be validated yet to reflect specifically nociception or opioid guidance with the current threshold values needs to be optimised for the use in routine care.

Previous studies have investigated the effect of opioid administration guided by nociception monitoring compared with a control group using clinical signs and have yielded conflicting results with regard to opioid consumption, intraoperative adverse events, and outcome parameters [20, 21].

It is controversial, whether nociception monitoring in general and SPI monitoring in particular reduces opioid consumption and improves postoperative recovery. Data on the impact of SPI guided opioid administration on the administered amount of opioid, postoperative short-term recovery, and long-term outcome have been inconclusive so far. Systematic reviews of randomised controlled trials investigated whether SPI guided anaesthesia affects opioid consumption [22–25]. Six reports compared SPI guided anaesthesia to standard practice and all six studies guided SPI values in the SPI guided group to maintain values below 50. Three of these six studies found that SPI monitoring reduced opioid consumption during surgery [26–28]. One study found an increased consumption of fentanyl in SPI guided patients [29]. In two studies there were no differences in intraoperative opioid consumption or perioperative secondary outcome parameters [30, 31]. In accordance with these meta-analyses, results of more recent studies were equally inconsistent: On the one hand, there are studies that found that SPI guided opioid administration resulted in higher opioid consumption compared to routine care, while other studies found equal consumption of remifentanil in both groups [21, 32, 33]. In this context, the present study is the first that investigated the effect of SPI guided opioid administration in patients with a combination of general anaesthesia and regional anaesthesia and the results could not demonstrate a benefit.

In general, many open questions remain with regard to the adequate intraoperative opioid dose. It remains unclear, if high or low doses of intraoperative opioids result in lower postoperative pain level. While there is at least weak evidence that high-dose intraoperative remifentanil administration increases pain scores in the post‐operative period, when compared with a low‐dose regimen, there is little evidence concerning non-remifentanil opioids [34]. A recent study with more than 61,000 patients found that reduced opioid administration during surgery resulted in an increase of postoperative pain and opioid consumption [35]. These findings question the paradigm that reducing intraoperative opioids results in a benefit for all patients with less postoperative pain and opioid demands. Thus, it is unclear if reducing intraoperative opioid consumption is a valuable goal for nociception monitoring.

The present study has some limitations. (1) First, our study pertains to patients with a combination of general anaesthesia and regional anaesthesia. Thus, our results are limited to this narrowly defined patient population and cannot be transferred to other patient populations. (2) Second, the study included different types of surgery which may have led to different forms of postoperative pain. This might have caused a bias in the measurements, considering the overall low sample size. (3) Despite the thorough randomisation the SPI guided group had a slightly higher body weight compared with the control group. Because the duration of surgery was equal in the study groups, there was a trend towards less sufentanil consumption per kg body weight and surgery duration in the SPI guided group while there was no difference between the study groups in the total sufentanil consumption. Still, this difference did not reach statistical significance. (4) Then, the study size was calculated for the co-primary endpoints. Hence, the study may be underpowered for detecting differences in the secondary endpoints. The results showed a trend towards less morphine equivalents administered 24 h after discharge from the PACU in the SPI guided group. Nevertheless, group comparisons were not adjusted for multiple testing and the study was not designed to proof this in a confirmatory manner. (5) Last, postoperative recovery is affected by numerous factors. Larger study populations are needed to differentiate between the effects from surgical trauma, intraoperative opioid titration and postoperative multimodal pain management for postoperative recovery.

## Conclusion

In conclusion, our study indicates that SPI guided sufentanil administration does not reduce intraoperative opioid administration, postoperative pain level and postoperative opioid consumption and does not enhance postoperative quality of recovery in patients with a combination of general and regional anaesthesia having trauma or orthopaedic surgery.

## Data Availability

No datasets were generated or analysed during the current study.
